# Randomized controlled trial demonstrates the benefit of RGTA^®^ based matrix therapy to treat tendinopathies in racing horses

**DOI:** 10.1371/journal.pone.0191796

**Published:** 2018-03-09

**Authors:** Sandrine Jacquet-Guibon, Anne-Gaelle Dupays, Virginie Coudry, Nathalie Crevier-Denoix, Sandrine Leroy, Fernando Siñeriz, Franck Chiappini, Denis Barritault, Jean-Marie Denoix

**Affiliations:** 1 Centre d’Imagerie et de Recherche sur les Affections Locomotrices Equines, Ecole Nationale vétérinaire d’Alfort, Maisons-Alfort, France; 2 Unité Sous Contrat 957, Biomécanique et Pathologie Locomotrice du Cheval, Institut National de la Recherche Agronomique, Ecole Nationale vétérinaire d’Alfort, Maisons-Alfort, France; 3 Clinique du Molinel, Marcq en Baroeul, France; 4 Episcience, London, United Kingdom; 5 Organ, Tissue, Regeneration, Repair and Replacement Société Actions Simplifiés, Paris, France; 6 Laboratoire de recherche sur la Croissance Cellulaire, Réparation, et Régénération Tissulaire, Faculté des Sciences, Université Paris-Est Créteil, Créteil, France; University of Pittsburgh, UNITED STATES

## Abstract

A randomized controlled trial was performed on racing horses, to evaluate the efficacy of a new class of therapeutic agents in regenerative medicine—ReGeneraTing Agents^®^ (RGTA^®^), to treat tendinopathies. Preliminary uncontrolled studies on tendon healing in racing horses with RGTA^®^ (OTR4131)—Equitend^®^ showed encouraging results, justifying performing a randomized, controlled, multicenter study with a two-year racing performance follow up. The objective of this study was to evaluate the effect of Equitend^®^ versus placebo on acute superficial digital flexor tendonitis in racing French Standardbred Trotters (ST). Twenty-two ST were randomly and blindly assigned to receive with a ratio of 2 to 1, a single Equitend^®^ (n = 14) or placebo (n = 8) intralesional injection under ultrasonographic guidance. Horses were evaluated over 4 months, by clinical and ultrasonographic evaluations (day 0, months 1, 2, 4), and their racing performances followed up over the 2 years after treatment. During the first month of treatment, a significant decrease in the cross-sectional area (CSA) was found in the Equitend^®^ group (p = 0.04). After 4 months, the number of Equitend^®^ treated horses with an improved CSA was significantly higher than the placebo-treated horses (p = 0.03571). The Equitend^®^ group returned to their pre-injury performance level, racing in, and winning, significantly more races than the placebo group (p = 0.01399 and 0.0421, respectively). Furthermore, recurrence was significantly higher in the placebo group than in the Equitend^®^ group (71.4% vs 16.6%, p = 0.02442). In conclusion, we measured a significant, short-term, reduction effect on CSA and demonstrated a long-term beneficial effect of intralesional injection of Equitend^®^ for the treatment of superficial digital flexor tendonitis on racing ST, racing 2. 3 times more often than placebo, with 3.3 times fewer recurrences maintaining pre-injury performance level. This study may open the way for the development of a human treatment of tendonitis.

## Introduction

Tendon injury remains a frequent condition in sport and race horses [1, 2], responsible for substantial wastage in the equine sports industry [3, 4], and justifying continuous development of research into new treatments. In the last decade, studies substantially focused on “biological therapies”, such as glycosaminoglycan polysulphate [5, 6], hyaluronic acid (HA) [5, 7], platelet rich plasma (PRP) [8–10], insulin-like growth factor-I [11], several sources of stems cells [12–17]. ReGeneraTing Agents (RGTA^®^) used in the present study belong to these “biological” or “nature mimicking” therapies. RGTA^®^ are nano-polysaccharides engineered by substituting dextran side chains with sulfate and carboxymethyl groups, to mimic heparan sulfates (HS). HS are a subset of glycosaminoglycans (GAGs), a key element in the extracellular matrix architecture, bridging and protecting matrix proteins, as well as storing, protecting and positioning heparan binding cytokines, chemokines and growth factors. Efficacy of RGTA^®^ on tissue repair was previously demonstrated in several experimental animal models and in human medicine in other tissues (bone, muscle, cornea, etc) [18–21]. These results triggered our interest to investigate the use of RGTA^®^ to treat horses’ tendinitis.

HS are degraded in injured tissue, due to inflammation mediated enzymatic degradation. The injected RGTA^®^ are resistant to enzymatic degradation, and replace HS at heparan-binding sites in the matrix, allowing the extracellular matrix scaffold and growth factors to reposition in the cellular microenvironment [18]. Equitend^®^ is an injectable formulation of RGTA^®^ OTR4131, which has been adapted specifically for the treatment of tendon injuries, by the addition of supplementary acetate groups which alters hydrophobicity [21]. Results of a previous preliminary clinical trial on 51 horses with superficial digital flexor tendonitis, treated with a single injection of Equitend^®^ [22] indicated an improvement of ultrasonographic appearance of tendinous lesion over the first 4 months and returned to racing 2.4 months earlier with higher level of earning (75% versus 15% in the matched controlled group). These encouraging results prompted us to design a double blind randomized, controlled multicenter clinical trial.

The objective of this clinical study was to evaluate the efficacy of Equitend^®^ (in terms of ultrasound and performance data) on superficial digital flexor (SDF) tendonitis in French Standardbred Trotters (ST) after a single intralesional injection of Equitend^®^ or placebo. The specific objectives were: i/ to compare the short term ultrasonographic healing of SDF tendonitis between groups receiving Equitend^®^ or placebo; ii/ to compare the racing performances of both groups with a follow-up period of two years after lesion.

## Materials and methods

### Trial design

A multicentric prospective controlled clinical trial was conducted at the Center of Imaging and Research on Equine Locomotor Injuries (CIRALE, Normandy, France) and in 2 equine practices in Normandy (Clinique Equine du Livet, 14 140 St Michel de Livet;- Clinique Equine de Méheudin, 61150 Echouché).

Institutional Animal Care and Use Committee of the National Veterinary School of Alfort (Ecole Nationale Vétérinaire d’Alfort, Maisons-Alfort, France) approved the study protocol.

Inclusion criteria for client-owned racing French Standardbred Trotters (ST) were clinical and ultrasonographic signs of unilateral, acute or subacute (less than one-mon duration) SDF tendonitis. Horses were only included if the client agreed to the study design and tendons had not received intralesional injections before. Concurrent lesion which could be considered as a predisposing factor of re-injury of the SDF tendon (*i*.*e*. lameness of the opposite limb), and bilateral lesions were exclusion criteria (S1 Fig and S1 Table).

Equitend^®^ treatment and placebo solutions were randomly assigned with a ratio of 2 treatments for one placebo (this ratio was chosen to improve client compliance and facilitate recruitment). Randomization procedure and blinding are described in S1 Fig. Briefly, Excel function (RANDBETWEEN(Bottom, Top)) was used to randomize the treatments between the two groups. Blinding was in place throughout the trial and revealed only once all data, including racing performances, were collected. None of the clinicians, experimenters, owners and trainers were aware of the content of the vials at any time.

### Clinical examination

Clinical examination included physical and dynamic evaluation. Thickening and heat of the metacarpal tendon area were evaluated as well as sensitivity to pressure of the SDF tendon on flexed limb, using a four-grade scale: 0 = no reaction at pressure, 1 = reaction to moderate pressure, 2 = reaction to mild pressure, 3 = violent reaction to mild pressure.

Dynamic evaluation was performed on straight line at trot and the grade of lameness was established according to the American Association of Equine Practitioners scoring system [23].

### Ultrasonographic examination

All horses underwent a complete ultrasonographic examination of both metacarpal tendon areas, using a high-resolution machine equipped with 7.5 MHz linear probe coupled with stand-off pad. Severity of the SDFT injury and treatment-associated changes were assessed by measuring the cross-sectional area (CSA) of the tendon at the maximal injured site (MIS), echogenicity, transverse lesion extent, transverse lesion architecture using the angle contrast ultrasound technique (ACUST) [24], longitudinal lesion extent and architecture according to the grading system presented in S2 Table. All the ultrasound images of each horse were scored blindly by four experienced veterinarians (JMD, AGD, SJ, VC) at once, at the end of the follow-up period. Time was not randomized, and images were reviewed and scored chronologically. The scores for all horses were made at the same time by each reviewer.

### Equitend^®^/Placebo treatment

**Fig 1:**
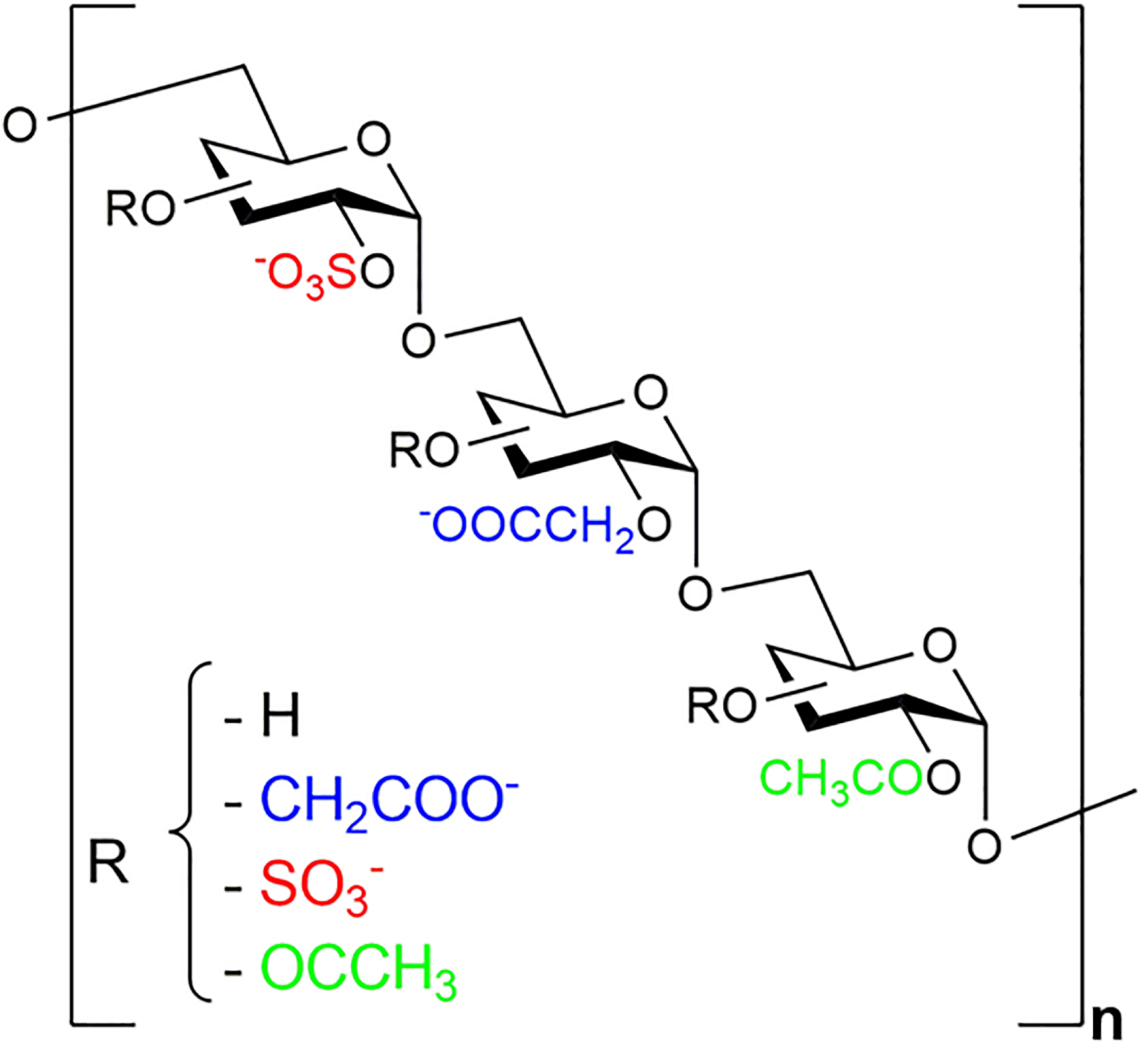
Chemical structure of Equitend^®^ active substance OTR4131. OTR4131 is an alpha 1–6 carboxymethyl sulfate acetate glucose polymer with a molecular weight ranged between 100 to 150 kDa.

Equitend^®^ product contained the GAG mimetic [OTR4131], obtained from OTR3 Inc. (Paris, France). OTR4131 is an alpha 1–6 polyglucose polymer substituted with carboxyl, sulphate and acetate groups as shown in Fig 1 [25], and prepared in 0.9% sodium chloride injectable form (0.15M NaCl), at the concentration of 10μg/mL Placebo vials were 0.9% sodium chloride only and absolutely identical to the Equitend^®^ vials.

After aseptic preparation of the tendon area, injection of 1ml of Equitend^®^ or placebo was performed on flexed limb, under ultrasound guidance at the level of the MIS, using a 0.5x16 mm needle (S1 Table) as previously described [22]. Horses were sedated by intravenous administration of detomidine (8 μg/kg) and butorphanol tartrate (16 μg/kg). Following injection, a light bandage was applied.

### Short-term follow-up and rehabilitation program

Clinical and ultrasonographic follow-up were performed at one (M_1_), two (M_2_) and four (M_4_) months after treatment or placebo injection using the same criteria as on inclusion day (S3 Table).

A standardized exercise program with interval training was recommended for all horses (S4 Table). This program was adjusted in accordance with the clinical and ultrasonographic findings and the expectations of the owner and trainer.

The use of a light shoe with wider toe and beveled branches for the front feet were recommended in all horses.

### Performance follow-up

All horses were followed up for two years after injury. The criteria used were: time to race post injury (established as the number of months from the treatment date to the first post-injury race), the number of races, the number of places, victories, the earnings per start post-injury. The results of performances after treatment were compared with results the year before injury. Racing data was obtained through websites (http://www.letrot.com/fr/fiche-horse and http://www.turf-fr.com/pack-pro). Re-injuries or end of careers were also recorded based on the information that the owners or the treating veterinarians gave us directly during the consultation or by phone call.

### Statistical analysis

All calculations were performed using R v.3.3.2 software [26].

S4 Table summarized the collected data at the corresponding time, and their format such as continuous, binary or ordinal variables. Because the variables did not follow a Gaussian distribution, we used non-parametrical tests to analyze the data. Using a non-parametric test avoids making any assumptions about the distribution of the variables (*i*.*e*. Gaussian distribution), the independency of the variables and the minimum number of observations required to perform the test such as in parametrical test. Therefore, Kruskal -Wallis test was used to analyze non-Gaussian variables and as a non-parametric method for testing whether groups originate from the same distribution, it was used for comparing two or more independent groups of equal or different sample sizes.

For the following tests, type I error-set is 5% (α = 5%).

#### Analysis of initial data

Baseline data at inclusion (D_0_, day of treatment) were described and compared between the treatment and placebo groups, using non-parametric Kruskal-Wallis test for continuous variables, chi-squared (χ^2^) test for binary variables, and Cochrane-Armitage test for ordinal variables. Chi-squared (χ^2^) test is the appropriate test to compare the distribution (*i*.*e*. frequency or proportion) between two or more groups such as recurrence. Finally, Cochrane-Armitage test is an extension of the chi-squared (χ^2^) test for the particular case when the categorical variable is ordinate (*i*.*e*. ordinal variable such as a grade from 0 to 4 as in ultra-sonographic variables) [27]. The test can be found in”coin” package in R-software [28].

#### Analysis of clinical and ultrasound data

In order to compare the clinical and ultrasonographic outcomes over time, the difference between data at the time-point considered (M_1_, M_2_ or M_4_) and baseline (M_0_ at D_0_) was calculated. Linear regression models were built in which the difference between M_i_ (i = 1, 2 or 4) and M_0_ (value at inclusion) of clinical and ultrasonographic variables were the dependent variables, and independent variables were treatment/placebo group, age at the individual level and the center at group-level. It provided an estimate of the difference between the two groups because of treatment (or treatment effect) for each time-period (M_0_ to M_1_, M_0_ to M_2_, and M_0_ to M_4_), adjusted on the horse age and inclusion centers (considered as fixed effects).

A generalized estimating equations (GEE) model was used for comparison of the cross-sectional area (CSA) between groups. To be closest to the normality, CSA was transformed in 1/(CSA)^2^. Horse age and center effect were introduced into the model as fixed-effect covariates [29].

Briefly, GEE is part of marginal models that do not require distributional assumptions for observations, only a regression model for the mean response. Marginal models provide a unified method for analyzing diverse types of longitudinal responses (*i*.*e*. continuous and ordinal variables), which avoids making assumptions about the distribution of the vector of responses; the method relies solely on assumptions about the mean response and the comparison of the coefficients and the variances [30].

#### Analysis of the performances variables

The performances such as earnings per race, number of races and number of victories before tendinitis (one year) and after treatment (*i*.*e*. 24-months after) were analyzed by using Kruskal-Wallis non-parametric chi-squared test. The recovery of the performances before tendinitis and after treatment was analyzed using the Wilcoxon signed rank pairwise non-parametric test with continuity correction.

The recurrence of tendinopathy over 2-years follow-up in both placebo and Equitend^®^-treated groups was assessed by Kaplan-Meier “survival” curve (“survival” R-package) and analyzed using Fleming-Harrington test [31, 32] because the effect of the therapy is more pronounced towards the middle and the end of the follow-up period (“survMisc” and “FHtest” R-packages). The recurrence over the 2-years after treatment was estimated by the odds ratio and the risk ratio using the small-sample adjusted method and compared by chi-squared test (“epitools” R-package) [33].

## Results

### Study population

Between May 2008 and February 2012, forty-two (n = 42) horses met the eligibility criteria, but only twenty-four (n = 24) ST presenting with acute (or subacute) SDF tendonitis in one front limb were included in the study because of lack of owners’ compliance for the eighteen (n = 18) horses not included (S1 Fig). Two horses were excluded during the follow-up period because they presented major colic signs (S1 Fig) that required administration of non-steroidal anti-inflammatory drugs. In the final 22 horses studied, there were 3 females, 8 males and 11 geldings. The mean age was 5.4 +/- 1.4 years old (range, 3 to 8 years). Horses were randomly assigned into Equitend^®^ (n = 14) or saline (n = 8) treatment groups and no significant differences in age, genders, duration of SDFT tendinopathies, affected forelimb, centers of investigation, lameness grades and sensitivity grades were observed between treatment groups (Table 1 and S1 Table). Hence all conditions were met for further investigation of the Equitend^®^ treatment.

**Table 1:** Characteristics of the study population at the date of inclusion.

	Placebo n = 8	Equitend^®^ Treatment (1 mL, OTR4131, 10 μg/mL) n = 14	*p-*value
**Age** (years, mean ± SEM)	5.3 ± 0.6	5.3 ± 0.4	0.09^‡^
**Gender** (F/G/M)	1/5/2	2/6/6	0.7^†^
**Duration of SDFT tendinopathy** before examination (days, mean ± SEM)	11.9 ± 2.9	25.8 ± 13.1	0.5^‡^
**Affected forelimb** R or L (n)	2/6	5/9	0.9^†^
**Centers of investigation** (C/CL/CM)	5/3/0	7/6/1	0.7^†^
**Lameness grades** n (%)	Grade 0: 2 (25)	Grade 0: 3 (21)	0.8^+^
	Grade 0.5: 0 (0)	Grade 0.5: 2 (14)	
	Grade 1: 4 (50)	Grade 1: 5 (36)	
	Grade 2: 2 (25)	Grade 2: 4 (29)	
**Sensitivity grades** n (%)	Grade 1: 3 (38)	Grade 1: 1 (7)	0.1^+^
	Grade 2: 2 (25)	Grade 2: 5 (36)	
	Grade 3: 3 (38)	Grade 3: 8 (57)	

Horses were randomly assigned into either Equitend^®^ treatment or placebo group. All horses included in this study are Standardbred trotter (ST). Comparison between placebo and Equitend^®^ by using ‡ unpaired *t*-test, † Chi-squared test with Yates’ correction, and + Cochrane-Armitage test for ordinal variables.

### Safety

As expected from preclinical safety and previous studies [22], intralesional injection was well tolerated for all horses and no adverse local or systemic effects were reported after treatment.

### Clinical and ultrasound follow-up

Analysis of clinical parameters on the day of inclusion (day 0) into the study revealed no significant difference between Equitend^®^ and saline treated groups. Ultrasonographic examination revealed an unexpected increase in the transversal lesion extent (p = 0.04) and a higher CSA at MIS (p = 0.01) in the Equitend^®^ treated group at day 0 (Table 2).

**Table 2:** Ultrasonographic and performance data at D0, and comparisons according to treatment groups.

Variables	Grade	Overall population (n = 22)	Control group (n = 8)	Treatment group (n = 14)	*p*-value
*Ultrasonographic variables*					
Echogenicity	2	3 (14%)	1 (12.5%)	2 (14%)	0.7
	3	12 (55%)	4 (50%)	8 (57%)	
	4	7 (32%)	3 (37.5%)	4 (29%)	
Transversal architecture	2	1 (5%)	0 (0%)	1 (7%)	0.08
	3	8 (36%)	3 (37.5%)	5 (36%)	
	4	13 (59%)	5 (62.5%)	8 (57%)	
Longitudinal architecture	2	2 (9%)	1 (12.5%)	1 (7%)	0.7
	3	14 (64%)	5 (62.5%)	9 (64%)	
	4	6 (27%)	2 (25%)	4 (29%)	
**Trans. Lesion extent**	1	2 (9%)	2 (25%)	0	**0.04**
	2	5 (23%)	3 (37.5%)	2 (14%)	
	2.5	2 (9%)	0 (0%°	2 (14%)	
	3	8 (36%)	2 (25%)	6 (43%)	
	4	5 (23%)	1 (12.5%)	4 (29%)	
Long. Lesion extent	3	2 (9%)	2 (25%)	0	0.3
	3.5	1 (5%)	0 (0%°	1 (7%)	
	4	19 (86%)	6 (75%)	13 (93%)	
**Cross Section Area—tendonitis side**		1.55 (1.27–1.77) 1.57 (0.37)	1.26 (1.14–1.45) 1.33 (0.32)	1.66 (1.13–1.88) 1.70 (0.34)	**0.01**
Cross Section Area—contralateral limb		0.85 (0.78–0.94) 0.88 (0.16)	0.79 (0.76–0.86) 0.80 (0.07)	0.87 (0.81–1.10) 0.93 (0.18)	0.08

Values are expressed as median (inter-quartile interval) and mean (standard deviation) for continuous variable, and as number (percent) for categorical variables.

While the CSA did not significantly change in the placebo treated group (p = 0.2), there was a significant decrease in CSA from M0 to M1 (from 1.70 to 1.61 cm^2^) in Equitend^®^ treated horses (p = 0.04, Tables 3 and 4) whereas in the placebo group CSA remained constant (from 1.33 to 1.34 cm^2^). These differences could be explained because in Equitend^®^-treated horses CSA was significantly higher compared to placebo group at the time of inclusion (M0) (p = 0.01).

**Table 3:** Treatment effect on cross section area (CSA) at the maximal injured site (MIS) on the affected limb in the period M_0_ to M_1_—General Estimating Equations (GEE) model.

Variables	Coefficient (95% CI)	*p*-value
Change of CSA in the control group between M_0_ and M_1_	-0.03 (-0.09 to 0.02)	0.2
Difference between the two groups at baseline -M_0_	-0.18 (-0.28 to -0.76)	**0.01**
Treatment effect on CSA between M_0_ and M_1_	0.07 (0.002 to 0.14)	**0.04**

CI: confidence interval.

**Table 4:** Mean CSA measures before and during the first four months of treatment.

	CSA mean ± SD cm^2^			
	M0	M1	M2	M4
Placebo (n = 8)	1.33 ±0.32	1.34 ± 0.16	1.29 ± 0.14	1.33 ± 0.17
Equitend (n = 14)	1.70 ± 0.34	1.61 ± 0.34	1.60 ± 0.36	1.63 ± 0.35

**Fig 2:**
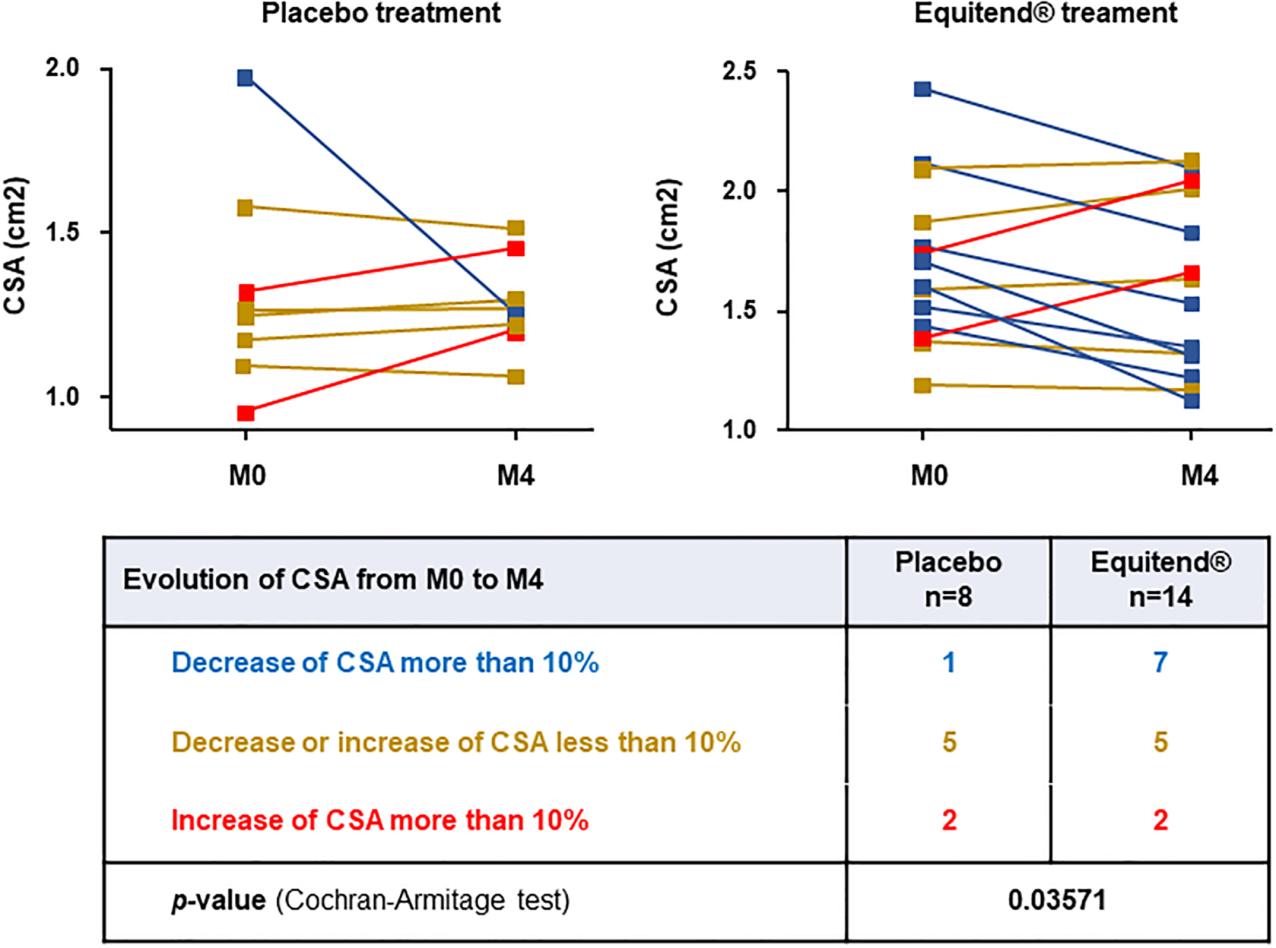
Improvement of CSA after 4 months of Equitend^®^ treatment. Graphics represent CSA (cm^2^) at the time of inclusion (M0) and after 4-months (M4) treatment for each horse in placebo group (left panel) and Equitend^®^-treated group (right panel). The table represent the number of horses in each treated group that improved more than 10% the CSA (■), not improved (■ Decrease or increase of CSA less than 10%), or worsened more than 10% (■). Data were compared by asymptotic Cochran-Armitage test.

Interestingly, the number of Equitend^®^-treated horses (n = 7) for which the CSA decreased more than 10%, after 4-months of treatment, was significantly higher (p = 0.03571) compared to the number of placebo-treated horses (n = 1, Fig 2). The average CSA improvement in Equitend^®^-treated horses (n = 7) was about 17% during this 4-months of treatment, with a CSA improvement in these 7 horses (Fig 2) ranged from an average of 1.80 cm^2^ (M0) to 1.50 cm^2^ (M4). In both groups, CSA did not change for 5 horses and deteriorated for 2 horses.

### Racing data analysis

Among the 22 included horses, 19 horses returned racing: 7 out of 8 horses (87.5%) in the placebo group and 12 out of 14 horses (85,7%) in the Equitend^®^-treated group. The three horses (one in placebo group and 2 in treated group) which did not resume racing, were retired for reasons unrelated to their tendonitis. The proportion between groups was not dissimilar (p = 0.974, chi-squared test). Analysis of performance in the year prior to the tendinopathies, such as number of races, number of victories and earnings per race did not show any differences between placebo and Equitend^®^-treated groups. Both groups are therefore considered as even.

Equitend^®^-treated horses raced significantly more than the placebo group (p = 0.01399) and the number of victories was significant higher (p = 0.0421, Table 5). Moreover, Equitend^®^-treated horses returned to their pre-injury performance level whereas placebo-treated horses did not (Table 5). In the placebo group the median number of races before treatment was 14 (interquartile [10–16]) whereas 24 months after injection it was 6 (interquartile [4.5–9.5], p = 0.0446). In the Equitend^®^-treated group, values before treatment were 15 [interquartile: 7.5–25] and became 15.5 after 24 months of treatment (interquartile [10–22.5], p = 0.6382).

**Table 5:** Equitend^®^-treated horses have better performances and recovery compared to placebo a year before tendinitis and over the two years after inclusion.

	12 months before treatment			24 months after treatment			Recovery (Before vs After)	
Performances of the horses	Placebo group n = 7	Treatment group n = 12	*p-value	Placebo group n = 7	Treatment group n = 12	*p-value	*Placebo* #*p*-value	*Treatment* #*p*-value
Earnings/race median [interquartile]	2845 [1484–6259]	2636 [1486–3090]	0.5617	439 [91–3131]	1296 [649–3302]	0.3525	0.1094	0.2593
Number of races median [interquartile]	14 [10–16]	15 [7.5–24.25]	0.5243	6.0 [4.5–9.5]	15.5 [10–22.5]	**0.01399**	**0.0446**	0.6382
Number of victories median [interquartile]	1 [1–2.5]	2 [0.75–3.5]	0.8293	0 [0–0]	1.5 [0–2]	**0.0421**	**0.0170**	0.2647

Values are expressed as median [inter-quartile range].

*p-values were calculated using the Kruskal-Wallis non-parametric chi-squared test.

^#^p-values were calculated using the Wilcoxon signed rank pairwise non-parametric test with continuity correction.

Similar results were observed for the number of victories. In the placebo group, no victories were reported after treatment, while the Equitend^®^-treated horses performed as before injury (Table 5).

**Fig 3:**
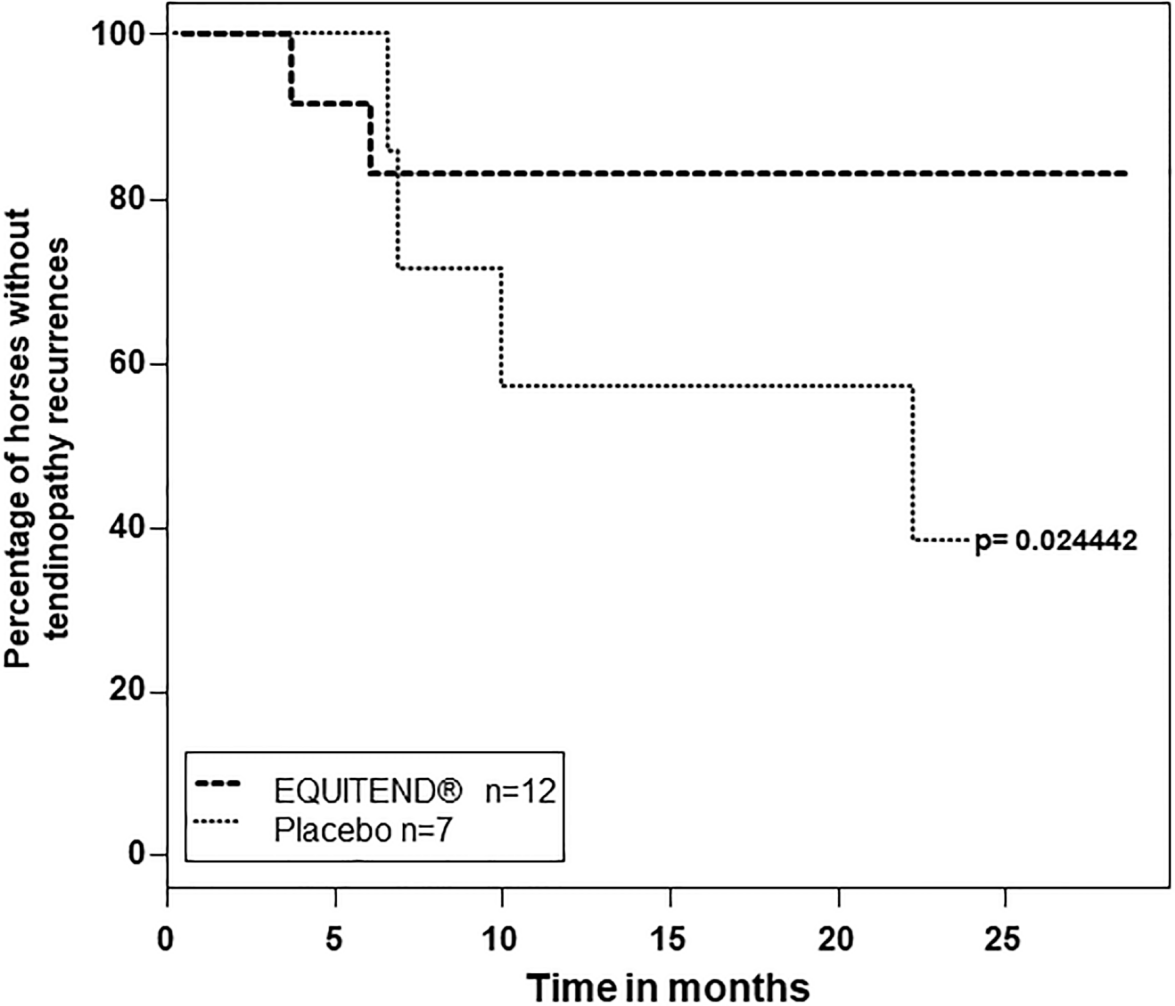
Equitend^®^-treated horses had significantly fewer recurrences of tendinopathy over 24-months follow-up. Kaplan-Meier curve shows recurrences of tendinopathy over 24-months follow-up of placebo (n = 7) and Equitend^®^-treated (n = 12) groups. p-value was estimated using Fleming-Harrington weights, emphasizing differences later in time. Fleming-Harrington is a Kaplan-Meier (product-limit) estimator.

The recurrence of tendinopathy over 24-months follow-up was assessed by Kaplan-Meier curve and showed that the placebo-treated group had significantly more recurrence of tendinopathy compared to Equitend^®^-treated group (Fig 3, p = 0.024442). Moreover, odds ratio (= 3.333) and risk ratio (= 2.476), p = 0.0671) are largely in favor of the Equitend^®^ group compared to the placebo group (Table 6).

**Table 6:** Placebo group has a higher risk of recurrence than Equitend^®^-treated group over the two years after inclusion.

Recurrence	Placebo group n = 7	Treatment group n = 12
** Yes**	4	2
** No**	3	10
**Odds ratio**	**3.333**	1
**Risk ratio**	**2.476**	1
**#p-value**	*0*.*0671*	

The odds ratio and the risk ratio are estimated by the small-sample adjusted method and # compared by Chi-square test

## Discussion

This study confirms efficacy of RGTA^®^ based matrix therapy in the treatment of tendinopathies. Sport and race horses are considered as a relevant model for humans over use tendonitis such as those developed in repeated motions and intense sport practice [34]. Therefore, the results presented here are expected to be supportive for a human efficacy study, although such a study needs to be designed and adapted. The reduced recurrence rate measured in this study is also a promising indicator to follow for human tendonitis. Another parameter associated with RGTA^®^ not reported in this study is pain relief observed in cornea [19, 35] and skin lesion [36]. For a human study, the pain reduction parameter would be of great interest.

Double blind, randomized, controlled, prospective clinical study is unanimously recognized as the gold standard for a clinical trial [37], however it is hard to recruit racing horses to reach a sufficient population to avoid any bias. This limit is due to the lack of owner compliance and represents a weakness of this study since the statistical analysis was limited by the small population. To our knowledge, we identified two field randomized clinical trials showing some efficacy of autologous MSC cells [13, 15]. Dyson (2004) was not able to perform a randomized placebo controlled trial because of this inherent client limitation [5]. To counteract these small groups a very homogeneous population with a detailed and long-term follow-up was studied. Another limit of the trial was the difference of lesion severity between the 2 groups at the time of inclusion, the CSA being significantly higher in the Equitend^®^ –treated group compared to the placebo group. This difference could not have been expected nor prevented as the randomization was blinded.

Indeed, CSA is the most objective measure of tendon pathology and can be assessed accurately using ultrasound for the tendinopathy follow-up, as extensively described before [38–40] but not necessarily directly related to the extent of the pathology.

Marr et al. (1993) identified that the severity of the lesions was related to the outcome on a population of 73 National Hunt racehorses [41]. It is reasonable to assume that difference between the 2 groups could have been more demonstrative in favor of the Equitend^®^ group if initial lesions were similar.

Most previous published data referred to retrospective [11], uncontrolled [13, 15, 16, 42] or matched [5, 9, 10, 22] trials. A study published by Smith et al. (2013), described a randomized and controlled clinical trial, to estimate the efficacy of autologous bone marrow stem cells on naturally occurring tendinopathy, but no performance follow-up was presented [43]. As the population studied was exclusively composed of retired National Hunt horses, the conclusion was based only on ultrasonographic healing, and results cannot be extrapolated to racehorses in activity. A recent prospective controlled clinical trial evaluated the effect of intralesional PRP treatment on naturally occurring SDF tendonitis [44]. The authors demonstrated that a higher proportion of treated horses were able to reach their previous or higher level of performance compared with placebo treated horses (80 versus 50% at 12 months, 60 versus 50% at 24 months). Nevertheless, the population studied was not homogeneous and only composed of sport and pleasure horses of various ages and levels of activity. To our knowledge the present study is the first prospective controlled clinical trial on racehorses with 2 years follow-up. According to the first Havemeyer workshop on equine tendon disease [37], evaluation of the efficacy of a treatment requires analysis of several objective outcome parameters (*i*.*e*. re-injury rate, proportion of horses racing more than 3 or 5 times).

Most clinical trials previously published were performed on flat and National Hunt racehorses or sport horses [2, 5, 10, 11, 16, 41, 43]. Few were made on Standardbreds [9, 11, 42, 45], and only three considered superficial digital flexor tendonitis [15, 42, 45]. The choice of ST in this study was based on the large population available in France, the easy accessibility to performance data, and the simple management of racing career (ST resume racing as soon as possible and can race all year long).

As described by Cheetham et al. (2010), several factors as age, sex, breed, discipline, gait, track surface (for Thoroughbred), could interfere with outcome [46]. In the present study, these parameters were very homogeneous between both studied groups. No difference of breed, sex and age, was identified, and trot is the only gait used in France. Furthermore, the placebo and Equitend^®^-treated groups also matched duration of SDFT tendinopathies, affected forelimb, centers of investigation, lameness grades and sensitivity grades.

Interestingly, echography analysis before treatment indicated a significant difference between the two groups. The Equitend^®^ group showed higher deterioration of the transectionnal lesion extent and cross-sectional area. After one month of treatment, no improvement was measured in the placebo group while the Equitend^®^-treated group showed significant improvement for both parameters.

CSA was significantly improved by 17% at M4 in 7 Equitend^®^ treated horses while only 1 horse in the placebo group spontaneously improved (p = 0.03571), suggesting that this improvement might have been even higher at 6 months as reported in other studies [16, 43, 47].

To ensure homogeneous conditions, the same rehabilitation program and shoeing were recommended for all horses. Activity was adapted according to the findings at ultrasound and clinical examinations as described above in “Short-term follow-up and Rehabilitation program” and S3 Table. A synthesis of the previously reported rehabilitation protocols was built on the 2007 Havemeyer symposium [37], but was dedicated to Thoroughbred (TB) racehorses or sport horses. Interestingly, although recovery under our treatment is faster, performance data registered were similar than those reported on TB [5, 10, 11, 13]. It would suggest that recovery period for SDF tendonitis on ST could be reduced compared to TB racehorses.

Emphasis was put on long-term analysis, with numerous performance data studied and up to 2 years follow-up after injury. Racing performances of the two populations were different: out of 8 Placebo treated horse 7 went back to racing but ran fewer times than previously whereas the Equitend^®^ group (12 out 14 horses) resumed similar activity and performances as before injury, running significantly (2.3 times) more than the placebo group. An average of four times higher recurrence was noted in the placebo group compared to Equitend^®^ treated group.

Dyson (2004) reported re-injury rates in various intralesional treatment groups with 42.5% for HA, 44.4% for PSGAG treatment or 45.5% (when including contralateral limb) for β-aminopropionitrile fumarate. Published data on the use of bone marrow mesenchymal stem cells showed lower re-injury rate with respectively 32.7% (when including contralateral limb) in Godwin et al. (2013) study [37, 43], and only 13% in Smith et al. (2013) study [37, 43]. In this last study, the re-injury rate was the unique outcome parameter studied, and analysis of several performance criteria is recommended to evaluate a treatment on tendonitis [37, 43]. In a study comparing performance of TB affected with SDF tendonitis matched with sound horses, O’Meara et al. showed that the assessment of the outcome of horses with SDF tendonitis using the number of races post injury required a minimum of 5 races post injury to be a useful indicator [4].

In the present study, a higher number of races post injury in the Equitend^®^ group was noted, with a higher rate of horses who competed more than 5 times. With respectively 78,6% and 64,3% of horses who competed more than 5 and 10 times on the Equitend^®^ group, these results compare favorably with our previous analysis for the same category of horses (Standardbreds) [42]. Hogan et al. (1995) published data on horses with desmotomy of the accessory ligament of the superficial digital flexor tendon (AL-SDFT), with a rate of 69% of horses racing more than 5 times [42]. Sawdon (1996) showed that 52% of horses competed more than 5 times with only conservative management [45]. In a more recent study, with use of autologous bone marrow aspirate alone or combined with desmotomy of the AL-SDFT, Russel et al. (2016) observed that 62% of treated horses raced more than 5 times and 53% more than 10 times [15].

Contrary to other treatments such as PRP, bone marrow or stem cells, Equitend^®^ is a stable, synthetic molecule. Moreover, only a single intralesional injection with a small volume of product was performed in this study. Therefore, Equitend^®^ could be considered by practitioners, as a more practical therapeutic option than PRP or stem cells.

More extensive studies should address the use of Equitend^®^ at earlier stage after tendinopathies injury as suggested by the mode of action of this molecule adapted to acute injury and to evaluate the benefit of a second injection in case of inconclusive ultrasonographic follow up data.

## Conclusion

Results of this study combined with the previous preliminary trial performed on Equitend^®^ demonstrate that a single intralesional injection of Equitend^®^ is safe and has beneficial effect for management of tendonitis in horses. Equitend^®^-treated horses recovered better then placebo treated horses, returning to a performance level similar to their pre-injury level.

Equitend is the first RGTA^®^ product developed to be injected for clinical uses and for a veterinary drug application, although many experimental tissue lesion animal models have demonstrated efficacy by injection. The results presented in this study will support development for a human use.

## Supporting information

S1 Fig Flow diagram of Equitend^®^ clinical trial. The figure summarized the steps followed to include the 24 horses on the clinical trial based on the consort recommendations (http://www.consort-statement.org/consort-statement/flow-diagram).
(PDF)

S1 Table Description and clinical history and treatment of each horse. Horses were randomly assigned into either Equitend^®^ treatment or placebo groups. Horses were sedated with detomidine (8 μg/kg) and butorphanol (16 μg/kg) and injection sites were prepared by shaving and sterilization. Equitend^®^ (1 mL, OTR4131, 10 μg/mL) or control saline solution was administered via intralesional injection under ultra-sonographic guidance into each injured tendon at the maximal injury site (MIS), using 25” gauge disposable sterile syringes. During the entire trial period, the composition of the solutions was known only by OTR3 to ensure blinding throughout the study. Following injection, a light bandage was applied. The horses were kept in stables for 24 h prior to their return to paddocks. Bandages were removed 24 h after injection and the injection site was monitored.
(PDF)

S2 Table Semi-qualitative and quantitative ultrasonographic criteria for characterizing the SDFT lesions. MIS: Maximal injury site; CSA: cross-sectional area; SDFT: superficial digital flexor tendon.
(PDF)

S3 Table Summary of the clinical, ultrasonography and performance variables, their measurement times, and format. *All data were collected for the horse, except the CSA that was collected for both side (tendonitis one and healthy counter-side). Meaning of the time-points: D_0_: inclusion date, and day of injection into the forelimb of the experimental treatment or placebo. M_1_, M_2_ and M_4_: repeated measurements of the variables. M_12_ and M_24_: for the calculation of number of race and victory(ies) 12 months before inclusion and 24 months after inclusion respectively.
(PDF)

S4 Table Guidelines for the rehabilitation program adjusted according to clinical and ultrasonographic findings at each control (M_1_, M_2_, M_4_). (PDF)

## Abbreviations

ACUST: angle contrast ultrasound technique
AL-SDFT: accessory ligament of the superficial digital flexor tendon
CSA: cross-sectional area
D: day
GAG: glycosaminoglycan
GEE: generalized estimating equations
HA: hyaluronic acid
HS: heparan sulfate
M: month
PRP: platelet-rich plasma
RGTA^®^: ReGeneraTing Agents^®^
SDF: superficial digital flexor
SDFT: superficial digital flexor tendon
ST: standardbred trotter
TB: thoroughbred

## References

[1] Clegg PD. Musculoskeletal disease and injury, now and in the future. Part 2: Tendon and ligament injuries. Equine Vet J. 2012;44(3):371–5. doi: 10.1111/j.2042-3306.2012.00563.x . 22486548 10.1111/j.2042-3306.2012.00563.x

[2] Kasashima Y, Takahashi T, Smith RK, Goodship AE, Kuwano A, Ueno T, et al Prevalence of superficial digital flexor tendonitis and suspensory desmitis in Japanese Thoroughbred flat racehorses in 1999. Equine Vet J. 2004;36(4):346–50. . 15163043 10.2746/0425164044890580

[3] Dowling BA, Dart AJ, Hodgson DR, Smith RK. Superficial digital flexor tendonitis in the horse. Equine Vet J. 2000;32(5):369–78. . 11037257 10.2746/042516400777591138

[4] O’Meara B, Bladon B, Parkin TD, Fraser B, Lischer CJ. An investigation of the relationship between race performance and superficial digital flexor tendonitis in the Thoroughbred racehorse. Equine Vet J. 2010;42(4):322–6. doi: 10.1111/j.2042-3306.2009.00021.x . 20525050 10.1111/j.2042-3306.2009.00021.x

[5] Dyson SJ. Medical management of superficial digital flexor tendonitis: a comparative study in 219 horses (1992–2000). Equine Vet J. 2004;36(5):415–9. . 15253082 10.2746/0425164044868422

[6] Moraes JR, Facco GG, Moraes FR, Engracia Filho JR, Miyazato LG, Beretta DC. Effects of glycosaminoglycan polysulphate on the organisation of collagen fibres in experimentally induced tendonitis in horses. Vet Rec. 2009;165(7):203–5. . 19684346 10.1136/vr.165.7.203

[7] Jann HW, Hart JC, Stein LE, Ritchey J, Blaik M, Payton M, et al The Effects of a Crosslinked, Modified Hyaluronic Acid (xCMHA-S) Gel on Equine Tendon Healing. Vet Surg. 2016;45(2):231–9. doi: 10.1111/vsu.12440 . 26767727 10.1111/vsu.12440

[8] Bosch G, van Schie HT, de Groot MW, Cadby JA, van de Lest CH, Barneveld A, et al Effects of platelet-rich plasma on the quality of repair of mechanically induced core lesions in equine superficial digital flexor tendons: A placebo-controlled experimental study. J Orthop Res. 2010;28(2):211–7. doi: 10.1002/jor.20980 . 19714688 10.1002/jor.20980

[9] Waselau M, Sutter WW, Genovese RL, Bertone AL. Intralesional injection of platelet-rich plasma followed by controlled exercise for treatment of midbody suspensory ligament desmitis in Standardbred racehorses. J Am Vet Med Assoc. 2008;232(10):1515–20. doi: 10.2460/javma.232.10.1515 . 18479242 10.2460/javma.232.10.1515

[10] Witte S, Dedman C, Harriss F, Kelly G, Chang YM, Witte TH. Comparison of treatment outcomes for superficial digital flexor tendonitis in National Hunt racehorses. Vet J. 2016;216:157–63. doi: 10.1016/j.tvjl.2016.08.003 . 27687944 10.1016/j.tvjl.2016.08.003

[11] Witte TH, Yeager AE, Nixon AJ. Intralesional injection of insulin-like growth factor-I for treatment of superficial digital flexor tendonitis in Thoroughbred racehorses: 40 cases (2000–2004). J Am Vet Med Assoc. 2011;239(7):992–7. doi: 10.2460/javma.239.7.992 . 21961641 10.2460/javma.239.7.992

[12] Caniglia CJ, Schramme MC, Smith RK. The effect of intralesional injection of bone marrow derived mesenchymal stem cells and bone marrow supernatant on collagen fibril size in a surgical model of equine superficial digital flexor tendonitis. Equine Vet J. 2012;44(5):587–93. doi: 10.1111/j.2042-3306.2011.00514.x . 22150794 10.1111/j.2042-3306.2011.00514.x

[13] Godwin EE, Young NJ, Dudhia J, Beamish IC, Smith RK. Implantation of bone marrow-derived mesenchymal stem cells demonstrates improved outcome in horses with overstrain injury of the superficial digital flexor tendon. Equine Vet J. 2012;44(1):25–32. doi: 10.1111/j.2042-3306.2011.00363.x . 21615465 10.1111/j.2042-3306.2011.00363.x

[14] Nixon AJ, Dahlgren LA, Haupt JL, Yeager AE, Ward DL. Effect of adipose-derived nucleated cell fractions on tendon repair in horses with collagenase-induced tendinitis. Am J Vet Res. 2008;69(7):928–37. doi: 10.2460/ajvr.69.7.928 . 18593247 10.2460/ajvr.69.7.928

[15] Russell JW, Russell TM, Vasey JR, Hall MS. Autologous bone marrow aspirate for treatment of superficial digital flexor tendonitis in 105 racehorses. Vet Rec. 2016;179(3):69 doi: 10.1136/vr.103620 . 27206445 10.1136/vr.103620

[16] Smith RK. Mesenchymal stem cell therapy for equine tendinopathy. Disabil Rehabil. 2008;30(20–22):1752–8. doi: 10.1080/09638280701788241 . 18608378 10.1080/09638280701788241

[17] Choi RK, Smith MM, Martin JH, Clarke JL, Dart AJ, Little CB, et al Chondroitin sulphate glycosaminoglycans contribute to widespread inferior biomechanics in tendon after focal injury. J Biomech. 2016;49(13):2694–701. doi: 10.1016/j.jbiomech.2016.06.006 . 27316761 10.1016/j.jbiomech.2016.06.006

[18] Barritault D, Desgranges P, Meddahi-Pelle A, Denoix JM, Saffar JL. RGTA(R)-based matrix therapy—A new branch of regenerative medicine in locomotion. Joint Bone Spine. 2016 . 27663756 10.1016/j.jbspin.2016.06.012

[19] Chebbi CK, Kichenin K, Amar N, Nourry H, Warnet JM, Barritault D, et al [Pilot study of a new matrix therapy agent (RGTA OTR4120) in treatment-resistant corneal ulcers and corneal dystrophy]. J Fr Ophtalmol. 2008;31(5):465–71. . 18641578 10.1016/s0181-5512(08)72462-8

[20] Desgranges P, Louissaint T, Allaire E, Godeau B, Kichenin K, Becquemin JP, et al First Clinical Pilot Study of Matrix Protection Therapy in Vascular Disease with Regenerating agent technology. Journal of Wound Technology. 2011;13:44–8.

[21] Mangoni M, Yue X, Morin C, Violot D, Frascogna V, Tao Y, et al Differential effect triggered by a heparan mimetic of the RGTA family preventing oral mucositis without tumor protection. Int J Radiat Oncol Biol Phys. 2009;74(4):1242–50. doi: 10.1016/j.ijrobp.2009.03.006 . 19545790 10.1016/j.ijrobp.2009.03.006

[22] Coudry V, Dupays A-G, Carnicer D, Jacquet S, Bertoni L, Crevier-Denoix N, et al Long-Term Follow-up of Superficial Digital Flexor Tendonitis Treated by a Single Intralesional Injection of a ReGeneraTing Agent in 51 Horses. Journal of Equine Veterinary Science. 2014;34(11):1357–60. doi: 10.1016/j.jevs.2014.09.014

[23] (AAEP) AAoEP. AAEP guidelines for reporting purchase examinations. 2009:6–7.

[24] Denoix JM, Bertoni L. The angle contrast ultrasound technique in the flexed limb improves assessment of proximal suspensory ligament injuries in the equine pelvic limb. Equine Veterinary Education. 2015;27(4):209–17. doi: 10.1111/eve.12303

[25] Sutton A, Friand V, Papy-Garcia D, Dagouassat M, Martin L, Vassy R, et al Glycosaminoglycans and their synthetic mimetics inhibit RANTES-induced migration and invasion of human hepatoma cells. Mol Cancer Ther. 2007;6(11):2948–58. doi: 10.1158/1535-7163.MCT-07-0114 . 18025279 10.1158/1535-7163.MCT-07-0114

[26] Team RC. R: A Language and Environment for Statistical Computing. R Foundation for Statistical Computing 2016.

[27] Wellek S, Ziegler A. Cochran-Armitage test versus logistic regression in the analysis of genetic association studies. Hum Hered. 2012;73(1):14–7. doi: 10.1159/000334085 . 22212245 10.1159/000334085

[28] Hothorn T, Hornik K, van de Wiel MA, Winell H, Zeileis A. Package "coin"-Conditional inference procedures in a permutation test framework. Repository CRAN. 2016;Version: 1.1–3:1–98.

[29] Lipsitz SR, Fitzmaurice GM, Orav EJ, Laird NM. Performance of generalized estimating equations in practical situations. Biometrics. 1994;50(1):270–8. . 8086610

[30] Gromping U. A note on fitting a marginal model to mixed effects log-linear regression data via GEE. Biometrics. 1996;52(1):280–5. . 8934596

[31] Oller R, Gomez G. A generalized Fleming and Harrington’s class of tests for interval-censored data Can J Stat. 2012;40(3):501–16.

[32] Harrington DP, Fleming TR. A class of rank test procedures for censored survival data. Biometrika. 1982;69(3):553–66. https://doi.org/10.1093/biomet/69.3.553.

[33] Heller D, Hoppe A, Restrepo S, Gatti L, Tournier AL, Tapon N, et al EpiTools: An Open-Source Image Analysis Toolkit for Quantifying Epithelial Growth Dynamics. Dev Cell. 2016;36(1):103–16. doi: 10.1016/j.devcel.2015.12.012 . 26766446 10.1016/j.devcel.2015.12.012 PMC4712040

[34] Lui PP, Maffulli N, Rolf C, Smith RK. What are the validated animal models for tendinopathy? Scand J Med Sci Sports. 2011;21(1):3–17. Epub 2010/08/03. doi: 10.1111/j.1600-0838.2010.01164.x . 20673247 10.1111/j.1600-0838.2010.01164.x

[35] Bata AM, Witkowska KJ, Wozniak PA, Fondi K, Schmidinger G, Pircher N, et al Effect of a Matrix Therapy Agent on Corneal Epithelial Healing After Standard Collagen Cross-linking in Patients With Keratoconus: A Randomized Clinical Trial. JAMA Ophthalmol. 2016;134(10):1169–76. Epub 2016/09/02. doi: 10.1001/jamaophthalmol.2016.3019 . 27584715 10.1001/jamaophthalmol.2016.3019

[36] Groah SL, Libin A, Spungen M, Nguyen KL, Woods E, Nabili M, et al Regenerating matrix-based therapy for chronic wound healing: a prospective within-subject pilot study. Int Wound J. 2011;8(1):85–95. Epub 2010/11/17. doi: 10.1111/j.1742-481X.2010.00748.x . 21078132 10.1111/j.1742-481X.2010.00748.x PMC7950993

[37] Smith RK, McIlwraith CW. Consensus on equine tendon disease: building on the 2007 Havemeyer symposium. Equine Vet J. 2012;44(1):2–6. doi: 10.1111/j.2042-3306.2011.00497.x . 22070097 10.1111/j.2042-3306.2011.00497.x

[38] Avella CS, Ely ER, Verheyen KL, Price JS, Wood JL, Smith RK. Ultrasonographic assessment of the superficial digital flexor tendons of National Hunt racehorses in training over two racing seasons. Equine Vet J. 2009;41(5):449–54. . 19642404 10.2746/042516409x391042

[39] Pickersgill CH, Marr CM, Reid SW. Repeatability of diagnostic ultrasonography in the assessment of the equine superficial digital flexor tendon. Equine Vet J. 2001;33(1):33–7. . 11191607 10.2746/042516401776767494

[40] Smith RK, Jones R, Webbon PM. The cross-sectional areas of normal equine digital flexor tendons determined ultrasonographically. Equine Vet J. 1994;26(6):460–5. . 7889919 10.1111/j.2042-3306.1994.tb04050.x

[41] Marr CM, Love S, Boyd JS, McKellar Q. Factors affecting the clinical outcome of injuries to the superficial digital flexor tendon in National Hunt and point-to-point racehorses. Vet Rec. 1993;132(19):476–9. . 8506599 10.1136/vr.132.19.476

[42] Hogan PM, Bramlage LR. Transection of the accessory ligament of the superficial digital flexor tendon for treatment of tendinitis: long term results in 61 standardbred racehorses (1985–1992). Equine Vet J. 1995;27(3):221–6. . 7556050 10.1111/j.2042-3306.1995.tb03066.x

[43] Smith RK, Werling NJ, Dakin SG, Alam R, Goodship AE, Dudhia J. Beneficial effects of autologous bone marrow-derived mesenchymal stem cells in naturally occurring tendinopathy. PLoS One. 2013;8(9):e75697 doi: 10.1371/journal.pone.0075697 . 24086616 10.1371/journal.pone.0075697 PMC3783421

[44] Geburek F, Gaus M, van Schie HT, Rohn K, Stadler PM. Effect of intralesional platelet-rich plasma (PRP) treatment on clinical and ultrasonographic parameters in equine naturally occurring superficial digital flexor tendinopathies—a randomized prospective controlled clinical trial. BMC Vet Res. 2016;12(1):191 Epub 2016/09/09. doi: 10.1186/s12917-016-0826-1 . 27604193 10.1186/s12917-016-0826-1 PMC5015224

[45] Sawdon H Y JV B T. Superficial digital flexor tendonitis in racehorses: long term follow up of conservatively managed cases. Australian Equine Veterinarian. 1996;14(1):21–5.

[46] Cheetham J, Riordan AS, Mohammed HO, McIlwraith CW, Fortier LA. Relationships between race earnings and horse age, sex, gait, track surface and number of race starts for Thoroughbred and Standardbred racehorses in North America. Equine Vet J. 2010;42(4):346–50. doi: 10.1111/j.2042-3306.2010.00032.x . 20525054 10.1111/j.2042-3306.2010.00032.x

[47] Muttini A, Russo V, Rossi E, Mattioli M, Barboni B, Tosi U, et al Pilot experimental study on amniotic epithelial mesenchymal cell transplantation in natural occurring tendinopathy in horses. Ultrasonographic and histological comparison. Muscles Ligaments Tendons J. 2015;5(1):5–11. . 25878980 PMC4396678

