# Supplementary material for: Randomized controlled trial demonstrates the benefit of RGTA® based matrix therapy to treat tendinopathies in racing horses
Source: PLoS One. 2018 Mar 9;13(3):e0191796. doi: 10.1371/journal.pone.0191796 (PMC5844532; doi:10.1371/journal.pone.0191796)
Supplement: S1 Fig — The figure summarized the steps followed to include the 24 horses on the clinical trial based on the consort recommendations (http://www.consort-statement.org/consort-statement/flow-diagram). (PDF) [file pone.0191796.s001.pdf]

Multi-centric Double Blinded Randomized Placebo Controlled Efficacy Clinical Trial
Approved by the Ethical Committee Com-Eth Afssa/Ecole Nationale Vétérinaire d'Alfort / University Paris-Est Creteil
File # 10-0030; Approval number 10/06/08-02B; Ammended 30/04/2009

Multi-centers (n= 3)
Time of eligibility May, 2008 to February, 2012

- CIRALE 14430 Goustranville.
- Clinique Equine du Livet, 14 140 St Michel de Livet ;
- Clinique Equine de Méheudin,61 150 Echouché.

Assessed for eligibility (n= 42)

- Sex and age: Indifferent
- Weight: Indifferent
- Breeds: Race horses (French Standardbred Trotters)
- Horse with a recent tendonitis on one forelimb superficial digital flexor tendon (SDFT). It is allowed if this is a repetition of an initial lesion
- Signing the consent form by the owner of the horse, or his/her representative

Horses non-eligible for inclusion (n= 18)
Lack of compliance of the owner

The non-inclusion criteria

- Horse with concomitant disease, which can be considered a predisposing factor for tendon injury recurrence (e. g. the contralateral limb lameness).
- Horse with SDFT tendonitis older than 4 weeks.
- Horse with SDFT tendonitis on the two forelimbs.
- Any Horse treated with anti-inflammatory drugs (steroids or not) for a period longer than 5 days at the time of inclusion
- Horses that are reformed after treatment without an attempt to return to the initial activity.

The exclusion criteria

- Any Horse which will be treated with anti-inflammatory drugs (steroids or not) for a period longer than 5 days. Note, the prescription of a treatment with anti-inflammatory drugs (steroids or not) not exceeding 5 days is allowed during the acute inflammatory phase of the tendonitis.
- Any horse whose ultrasound monitoring at Day 30 and Day 60 will not be realized.
- Exclusion can only be decided by the principal investigator (Prof JM Denoix) and be contained in a record provided for this purpose

Randomized (n= 24, ratio 2/1, 3 institutions)
Time of inclusion May 7th, 2009 to June 1st, 2011

Randomization:
- The 24 horses included in the clinical trial will be randomized into 2 groups:
- A group of 16 horses (2 thirds): the treatment group 'S' receiving EQUITEND® treatment.
- The other 8 horses (or 1 third): the control group 'P' receiving placebo (saline solution).

The randomization list has been prepared by OTR3 and only known by the person who prepares the doses.
The randomization information was confidential and secured during the entire duration of the study.
- 3 institutions (k=3)
- 3 blocks of 8
- Excel function to assigned numbers for treatment (RANDBETWEEN(Bottom;Top))

Masking:
- Personnel responsible for injection and assessments were not aware of the nature of the injected product and of the allocation of animals to the different treatment groups.

Allocated to EQUITEND-treatment (n= 16)
- Test item at 10 µg/mL of OTR4131
- Containers: vials of 1.0mL
- Description: sterile "ready-to-use" solution (OTR4131, diluted in 0.9% NaCl) (without preservatives or any other additions) packaged in a glass bottle with a bromobutyl stopper and sealed by an aluminum capsule.
- Storage at room temperature

Excluded (n= 2)
- Major colic signs

Lost to follow-up (give reasons) None

Clinical data Analyzed (n= 14)
- All data recorded

Excluded from analysis (n= 2)
no racing again
no performances recorded
no winnings recorded
no recurrence recorded

Analysis of the Performances (n= 12)

Allocated to Placebo-treatment (n= 8)
- Test item: 0.9% NaCl
- Containers: vials of 1.0mL
- Description: sterile "ready-to-use" solution (without preservatives or any other additions) packaged in a glass bottle with a bromobutyl stopper and sealed by an aluminum capsule.
- Storage at room temperature

Lost to follow-up (give reasons) None

Clinical data Analyzed (n= 8)
- All data recorded

Excluded from analysis (n= 1)
no racing again
no performances recorded
no winnings recorded
no recurrence recorded

Analysis of the Performances (n= 7)

S1 Fig. Flow diagram of Equitend® clinical trial. The figure summarized the steps followed to include the 24 horses on the clinical trial based on the consort recommendations (http://www.consort-statement.org/consort-statement/flow-diagram).
