# Supplementary material for: Randomized controlled trial demonstrates the benefit of RGTA® based matrix therapy to treat tendinopathies in racing horses
Source: PLoS One. 2018 Mar 9;13(3):e0191796. doi: 10.1371/journal.pone.0191796 (PMC5844532; doi:10.1371/journal.pone.0191796)
Supplement: S1 Table — Horses were randomly assigned into either Equitend® treatment or placebo groups. Horses were sedated with detomidine (8 μg/kg) and butorphanol (16 μg/kg) and injection sites were prepared by shaving and sterilization. Equitend® (1 mL, OTR4131, 10 μg/mL) or control saline solution was administered via intralesional injection under ultra-sonographic guidance into each injured tendon at the maximal injury site (MIS), using 25” gauge disposable sterile syringes. During the entire trial period, the composition of the solutions was known only by OTR3 to ensure blinding throughout the study. Following injection, a light bandage was applied. The horses were kept in stables for 24 h prior to their return to paddocks. Bandages were removed 24 h after injection and the injection site was monitored. (PDF) [file pone.0191796.s002.pdf]

S1 Table. Description and clinical history and treatment of each horse.

Number	Breed	Gender	Age (Years)	Duration of SDFT tendinopathy until initial examination	Affected forelimb	Treatment	Centre
1	ST	Gelding	5	16 d	L	Equitend®	C
2	ST	Gelding	6	11 d	L	Equitend®	C
3*	ST	Female	5	14 d	L	Equitend®	C
4*	ST	Gelding	4	20 d	R	Equitend®	C
5	ST	Female	6	9d	R	Equitend®	CL
6	ST	Male	6	21 d	R	Equitend®	CL
7	ST	Gelding	4	18 d	L	Equitend®	CL
8	ST	Male	7	11 d	R	Equitend®	CM
9	ST	Male	3	6 months	L	Equitend®	C
10	ST	Male	5	8 d	L	Equitend®	C
11	ST	Male	4	-	R	Equitend®	C
12	ST	Female	8	0 d	R	Equitend®	C
13	ST	Gelding	8	15 d	L	Equitend®	CL
14	ST	Gelding	7	21 d	L	Equitend®	CL
15	ST	Gelding	6	0 d	L	Equitend®	CL
16	ST	Male	5	-	L	Equitend®	C
17	ST	Male	3	3 d	L	saline	C
18	ST	Gelding	4	2 d	R	saline	C
19	ST	Gelding	5	-	L	saline	C
20	ST	Gelding	4	14 d	R	saline	CL
21	ST	Gelding	2	23 d	L	saline	CL
22	ST	Gelding	7	10 d	L	saline	C
23	ST	Male	5	10 d	L	saline	CL
24	ST	Female	6	21 d	L	saline	C

Horses were randomly assigned into either Equitend® treatment or placebo groups. Horses were sedated with detomidine (8 µg/kg) and butorphanol (16 µg/kg) and injection sites were prepared by shaving and sterilization. Equitend® (1 mL, OTR4131, 10 µg/mL) or control saline solution was administered via intralesional injection under ultra-sonographic guidance into each injured tendon at the maximal injury site (MIS), using 25" gauge disposable sterile syringes. During the entire trial period, the composition of the solutions was known only by OTR3 to ensure blinding throughout the study. Following injection, a light bandage was applied. The horses were kept in stables for 24 h prior to their return to paddocks. Bandages

were removed 24 h after injection and the injection site was monitored. * This horse was discarded from the study because it developed major colic signs. C: CIRALE; CL: Clinique Equine du Livet; CM: Clinique Equine de Méheudin.
