# Supplementary material for: Randomized controlled trial demonstrates the benefit of RGTA® based matrix therapy to treat tendinopathies in racing horses
Source: PLoS One. 2018 Mar 9;13(3):e0191796. doi: 10.1371/journal.pone.0191796 (PMC5844532; doi:10.1371/journal.pone.0191796)
Supplement: S2 Table — MIS: Maximal injury site; CSA: cross-sectional area; SDFT: superficial digital flexor tendon. (PDF) [file pone.0191796.s003.pdf]

S2 Table. Semi-qualitive and quantitative ultrasonographic criteria for characterizing the SDFT lesions

Criteria	Evaluated on	Grades
**Echogenicity**	*Transverse section at MIS*	**0**: normal echogenicity (sound tendon)<br>**1**: mild change of echogenicity ("more bright spots than dark spots") = mild hypoechogenicity<br>**2**: moderate change of echogenicity ("same amount of bright and dark spots") = hypoechogenicity<br>**3**: severe change of echogenicity ("less bright spots than dark spots") = marked hypoechogenicity<br>**4**: anechogenicity
**Size of the lesion = Extent**	*Transverse section at MIS*	**0**: sound tendon<br>**1**: lesion <10 % of the CSA of the SDFT<br>**2**: lesion >10%, <25% of the CSA of the SDFT<br>**3**: lesion >25%, <50% of the CSA of the SDFT<br>**4**: lesion >50% of the CSA of the SDFT
	Longitudinal section	**0**: sound tendon<br>**1**: length of the lesion < 1cm<br>**2**: length of the lesion > 1cm and < 2cm<br>**3**: length of the lesion > 2cm and < 4cm<br>**4**: length of the lesion > 4cm
**Architecture**	*Transverse section at MIS*	**0**: normal architecture (sound tendon) = echogenic peritendon<br>**1**: lesion is echogenic on positive image and hypoechogenic on negative image (good healing, without fibrous tissue)<br>**2**: lesion is echogenic on positive image and echogenic on negative image (fibrous scar tissue)<br>**3**: lesion is hypoechogenic on positive image and echogenic on negative image (mixture of fibrous tissue with fibroplasia, granulation tissue or disrupted fibres)

		**4**: lesion is hypoechogenic on positive image and hypoechogenic on negative image (initial lesion or re-injury)
	Longitudinal section	**0**: normal architecture (sound tendon)<br>**1**: mild alteration of fibre pattern (most of the fibres are parallel, dense echogenic fibres)<br>**2**: moderate alteration of fibre pattern (as many parallel fibres as disrupted fibres, short echogenic fibres)<br>**3**: severe alteration of the fibre pattern (a few fibres are parallel, short hypoechogenic fibres)<br>**4**: complete disruption of fibre pattern (area without fibre pattern)
**CSA (cm^2)**	*Transverse section at MIS*	**Two successive measures that had to differ from less than 5%.** If difference of more than 5% was noted, the measurement was repeated until two comparable measures were obtained.

MIS: Maximal injury site; CSA: cross-sectional area; SDFT: superficial digital flexor tendon.
