# Supplementary material for: Randomized controlled trial demonstrates the benefit of RGTA® based matrix therapy to treat tendinopathies in racing horses
Source: PLoS One. 2018 Mar 9;13(3):e0191796. doi: 10.1371/journal.pone.0191796 (PMC5844532; doi:10.1371/journal.pone.0191796)
Supplement: S3 Table — *All data were collected for the horse, except the CSA that was collected for both side (tendonitis one and healthy counter-side). Meaning of the time-points: D0: inclusion date, and day of injection into the forelimb of the experimental treatment or placebo. M1, M2 and M4: repeated measurements of the variables. M12 and M24: for the calculation of number of race and victory(ies) 12 months before inclusion and 24 months after inclusion respectively. (PDF) [file pone.0191796.s004.pdf]

S3 Table. Summary of the clinical, ultrasonography and performance variables, their measurement times, and format

Variables		Time of measurement					
**Name**	**Format**	**M-12**	**D0**	**M1**	**M2**	**M4**	**M24**
Clinical variables							
Age	Continuous		x				
Sensibility-Reactivity grade	Ordinal (0 to 3)		x	x	x	x	
Lameness	Ordinal (0 to 5)		x	x	x	x	
Ultrasonography variables							
Echogenicity	Ordinal (0 to 4)		x	x	x	x	
Trans. architecture	Ordinal (0 to 4)		x	x	x	x	
Long. architecture	Ordinal (0 to 4)		x	x	x	x	
Trans. lesion extent	Ordinal (0 to 4)		x	x	x	x	
Long. lesion extent	Ordinal (0 to 4)		x	x	x	x	
Cross Section Area (CSA)*	Continuous		x	x	x	x	
Performance variables							
Mean earnings/race	Continuous	x					x
Time inclusion back to race	Continuous						
Number of races	Continuous	x					x
Number of victories	Continuous	x					x
Recurrence	Binary and date						x

*All data were collected for the horse, except the CSA that was collected for both side (tendonitis one and healthy counter-side).

Meaning of the time-points:

- D_0: inclusion date, and day of injection into the forelimb of the experimental treatment or placebo.
- M_1, M_2 and M_4: repeated measurements of the variables.
- M_{-12} and M_{24}: for the calculation of number of race and victory(ies) 12 months before inclusion and 24 months after inclusion respectively.
