# Supplementary material for: Randomized controlled trial demonstrates the benefit of RGTA® based matrix therapy to treat tendinopathies in racing horses
Source: PLoS One. 2018 Mar 9;13(3):e0191796. doi: 10.1371/journal.pone.0191796 (PMC5844532; doi:10.1371/journal.pone.0191796)
Supplement: S4 Table — (PDF) [file pone.0191796.s005.pdf]

S4 Table. Guidelines for the rehabilitation program adjusted according to clinical and ultrasonographic findings at each control (M_1, M_2, M_4)

Months	Duration and nature of the exercise
1	Rest with 15 mins hand walking exercise twice daily
2	Warm-up 20 mins at walk<br>Interval training with 3-5 fractions (100-300m) at 15km/h
3	Warm-up 20 mins at walk<br>Interval training with 5-8 fractions (300m) at ≤20km/h
4	Warm-up 20 mins at walk<br>Interval training with 2-3 fractions (1000m) at ≤25km/h
5	Warm-up 20 mins at walk<br>Interval training with 3-4 fractions (1000m) at ≤30km/h (2'/km)
6	Warm-up 20 mins at walk<br>Interval training with 2-3 fractions (2000m) at ≤36km/h (1'40/km)
7-8	Back to full work
9-10	Back to race
